# A Novel Indirect-Direct Technique for Provisional Fixed Partial Denture Fabrication in Nonparallel Abutment Cases

**DOI:** 10.7759/cureus.87286

**Published:** 2025-07-04

**Authors:** Keerthi Rohini, Tushar Tanwani, Sudeepti Soni, Shruti Jumde, Anindita Chakraborty

**Affiliations:** 1 Department of Prosthodontics and Crown and Bridge, New Horizon Dental College and Research Institute, Bilaspur, IND

**Keywords:** fixed partial dentures, fixed prosthesis, non-parallel abutments, precision attachment, provisional restoration

## Abstract

Fabrication of provisional fixed partial dentures in cases with nonparallel abutments presents a unique prosthodontic challenge, especially when esthetic and hygienic demands must be balanced with mechanical retention. Traditional approaches often utilize visible clasps or rely on non-esthetic stabilizing components that compromise patient comfort and appearance. This technical note describes an innovative technique that eliminates the need for clasps by incorporating a triangular rest seat with a dovetail configuration, designed to provide multidirectional stability. Reinforcement within the rest seat and sealing with composite resin ensures a clean, esthetic finish while maintaining the structural integrity and retention of the prosthesis. In addition, a segmental fabrication approach allows for precise adaptation around nonparallel abutments. This technique offers a conservative, patient-friendly solution that improves esthetics, hygiene, and functional reliability in provisional restorations.

## Introduction

Provisional fixed partial dentures (FPDs) serve a crucial role in prosthodontics by maintaining occlusal stability, preserving soft tissue contours, supporting esthetics and phonetics, and allowing for patient adaptation during the interim phase of treatment [1]. Beyond these essential functions, provisional restorations offer valuable diagnostic insights into occlusal dynamics and prosthesis design prior to definitive rehabilitation.

A critical factor influencing the success of a provisional FPD is the alignment of abutment teeth, the natural teeth on either side of the edentulous space that support the prosthesis. Ideally, these abutments should be convergent, meaning their preparation surfaces lean slightly toward each other, facilitating a common path of insertion and enhancing mechanical retention. By contrast, divergent abutments, where the teeth lean away from each other, pose a significant clinical challenge. This lack of parallelism between abutments complicates prosthesis fabrication, as it hampers proper seating, reduces retention, and increases the risk of displacement or rocking during function [2].

To address such complications, traditional methods have included precision attachments, mechanical clasps, aggressive path-modifying tooth preparations, or multi-part sectional prostheses [2-4]. Although effective, these approaches are not without drawbacks. For instance, the use of metal clasps may compromise esthetics, particularly in the anterior or premolar regions, and may also promote plaque accumulation and gingival irritation over time [5,6].

In efforts to improve stability while preserving esthetics, some clinicians have explored the use of rest seats, small depressions prepared in the occlusal or proximal surfaces of crowns or teeth to receive part of the prosthesis for support. A commonly used design is the triangular-shaped rest seat, which offers vertical support [7]. However, such designs alone often fail to control lateral (side-to-side) or proximal (mesial-distal) displacement, especially in cases of pronounced abutment misalignment [8]. Moreover, they rarely incorporate concealed reinforcement mechanisms that can enhance both retention and durability without compromising the aesthetic outcome.

Recognizing these limitations, this technical note presents an innovative indirect-direct technique for fabricating a two-part, non-sectioned provisional FPD that avoids visible clasping. The technique employs a rest seat with a dovetail configuration, which is a flared internal design that helps lock the prosthesis in place, which was integrated into the provisional crown on one abutment. This is internally reinforced using wrought wire and sealed with composite resin to maintain a seamless, hygienic appearance. This approach provides a conservative, patient-friendly solution for managing nonparallel abutments, particularly when re-preparation is not feasible or when esthetic demands preclude conventional retentive designs.

## Technical report

**Figure 1:**
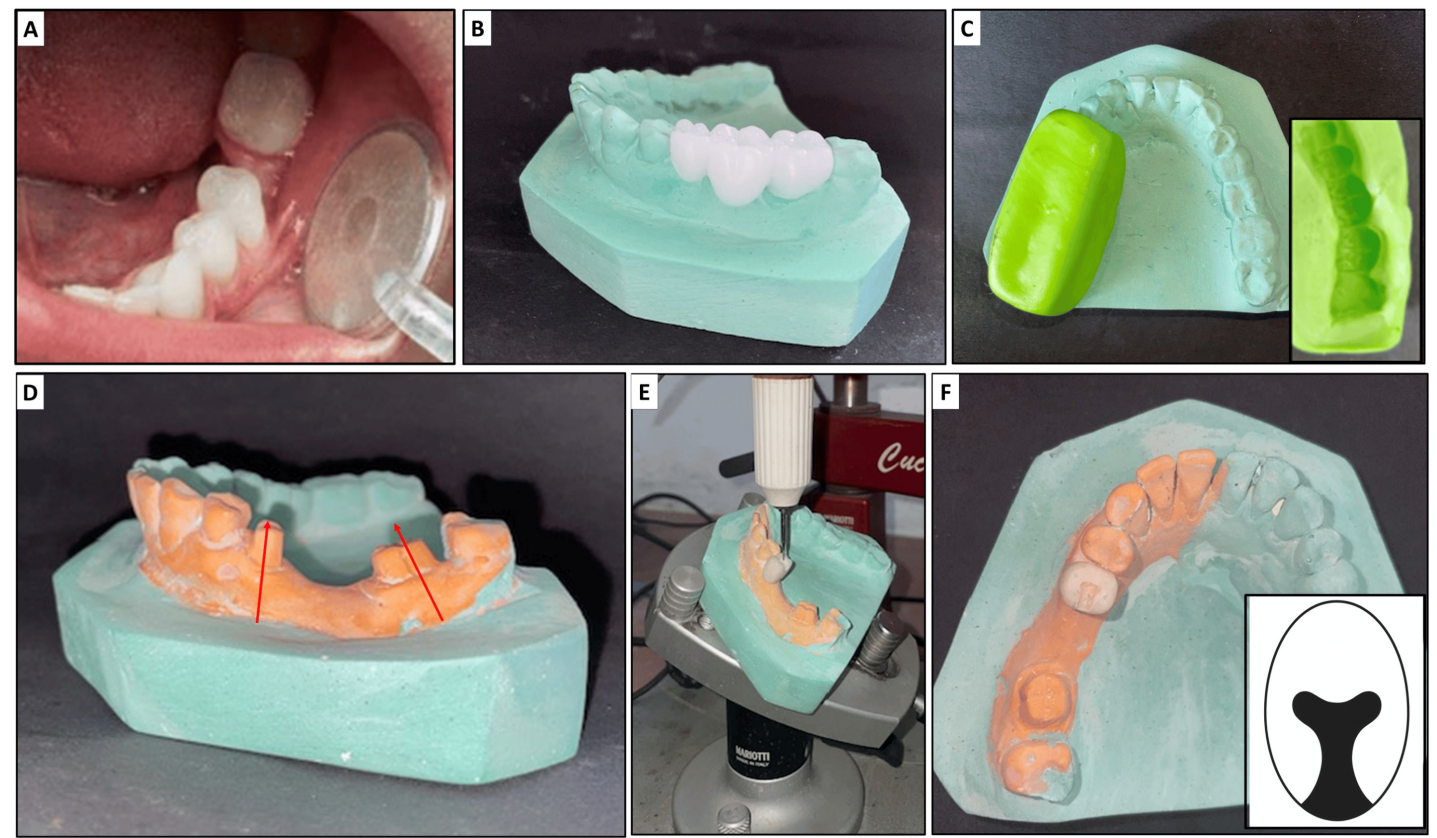
A) Tilted mandibular second premolar and second molar; B) wax up with teeth 35, 36, 37; C) putty index on the primary cast (inset: impression surface of the putty index); D) diagnostic cast showing prepared abutment teeth (lines indicate non-parallelism) ; E) preparation of the rest seat on the provisional crown fabricated on the tooth 35; F) prepared rest seat (inset: diagrammatic representation of the dovetail design).

Consider a case of a missing mandibular left first molar with a tilted mandibular second premolar and second molar adjacent to the space (Figure 1A). Make two diagnostic impressions of the mandibular arch, one of which will be used for the diagnostic wax-up and the other for cast modification and provisional fabrication. Pour the first impression to obtain a diagnostic cast. Establish a proper occlusal relationship and perform a full-contour wax-up (Figure 1B) in the edentulous region and adjacent abutments to simulate the final prosthetic design. Fabricate a putty index (Figure 1C) of the wax-up using a silicone putty material (3M ESPE ExpressTM XT, India) to accurately capture the morphology of the waxed units (35, 36, and 37 in this case).

On the second cast, prepare the abutment teeth for a porcelain-fused-to-metal crown (35 and 37, for porcelain-fused-to-metal bridge in this case) conservatively, ensuring minimal reduction (Figure 1D). As noted on the cast, no changes were made to the third molar. These preparations are more conservative than the actual intraoral tooth preparations planned later, as this is a diagnostic indirect-direct technique. Conservative tooth preparation on the diagnostic cast was performed to guide provisional fabrication while preserving tooth structure, allowing flexibility for intraoral adjustments during final preparation. This approach ensures biological preservation and optimizes the path of insertion planning in the indirect-direct technique.

In this case, a tapered fissure bur was used, and a shoulder margin was given with a flat tapered bur. The occlusal surface reduction was 1.5 mm in functional cusps and 1 mm for non-functional cusps. The proximal surface reduction was also 1 to 1.5 mm. A deep chamfer or shoulder finish line can be given, if porcelain is needed for esthetics. A taper of 6°-10° total occlusal convergence (3°-5° per wall) was given to allow a single path of insertion.

Apply a separating medium to the prepared cast surfaces. Mix self-cure provisional acrylic resin (PYRAX self-cure 10, India) as per the manufacturer’s instructions and load it into the putty index. Seat the index over the prepared area corresponding to the abutment tooth, which is relatively more vertical in position (35 in this case). Once the material sets, remove the index, trim the excess, and polish the provisional crown. When the patient’s oral hygiene is excellent, and in the case of well-healed broad residual ridges, we can go for tissue contact pontic along with a hygienic pontic.

Prepare a triangular rest seat of 1 to 1.5mm depth on the provisional crown to prevent lateral displacement (Figure 1E). Shape the mesial wall of the rest seat to be nearly parallel to the path of insertion of the other non-parallel abutment tooth (37 in this case). Incorporate a dovetail form into the rest seat to prevent the horizontal displacement of the attachment system (Figure 1F). The steps up to this stage are collectively depicted in Figure 1.

**Figure 2:**
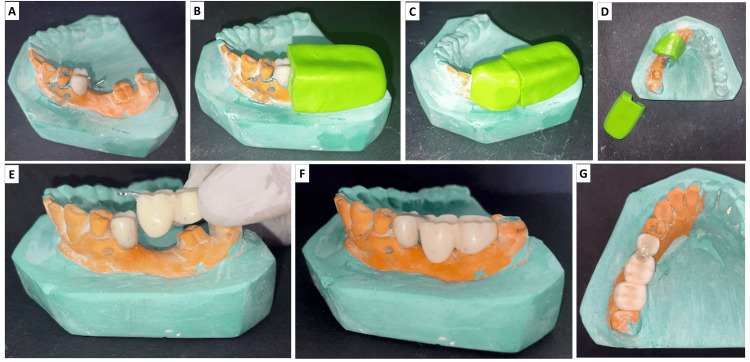
A) Securing clasp on the rest seat of provisional crown on 35; B), C), D) a wrought wire was secured on to the rest seat with the help of the putty index and the second part of the provisional FPD was fabricated; E) second part provisional FPD associated with teeth 36 and 37; F) a wrought wire was reinforced with a bis-GMA composite; G) occlusal view of two-part provisional FPD with teeth 35, 36, and 37. FPD: fixed partial denture

Customize a 22-gauge stainless steel wrought wire to match the contour of the prepared rest seat (Figure 2A). Stabilize the wire using a putty index to hold it securely in position and prevent dislodgement during subsequent steps (Figure 2B-D). Load fresh provisional acrylic resin into the same putty index and seat it over the unprepared regions on the cast (36 and 37 in this case). This allows the resin to engage and lock the loops of the previously adapted wrought wire into the provisional crown of 36. To avoid bonding of fresh acrylic to the acrylic crown on 35, always use a separating medium or physical barrier before adding new acrylic. This simple step ensures your prosthesis segments remain separate and function as intended, making adjustments and removals much easier.

After polymerization, remove the index and finish and polish the segment. Reinforce the interface of the rest seat on the provisional crown by sealing and securing the wrought wire in place using bis-GMA composite. If the provisional crown became dislodged or broke, it was remade and re-cemented to maintain function and aesthetics. The patient wore the provisional prosthesis for a period sufficient to evaluate fit, function, and tissue response before the final prosthesis was cemented. The provisional crown was in place for approximately four weeks before the final prosthesis was delivered. Prepare the abutment teeth intraorally as per clinical requirements. Try in the two-part provisional FPD intraorally (Figure 2E). Evaluate the fit and path of insertion, and adjust as necessary (Figure 2F, 2G). If any discrepancies in adaptation are noted, particularly at the margins or tissue surface, reline the internal aspect using a chairside reline material to ensure optimal adaptation to the prepared teeth. After relining, verify and adjust occlusal contacts in centric and excursive movements to eliminate any premature contacts before final cementation of the provisional restoration. The procedures are collectively summarized in Figure 2.

If the provisional crown became dislodged or broke, it was remade and re-cemented to maintain function and aesthetics. The patient wore the provisional for a period sufficient to evaluate fit, function, and tissue response before the final prosthesis was cemented. According to the case report, the provisional was in place for approximately four weeks before the final prosthesis was delivered.

## Discussion

Managing nonparallel abutments during provisional FPD fabrication remains a notable challenge in prosthodontic practice. Traditional solutions often require mechanical components such as clasps or auxiliary rests to compensate for the lack of a common path of insertion [2-4]. While functional, these strategies frequently compromise esthetics, plaque control, and patient comfort.

Although the abutments in the present case are convergent rather than divergent, the decision to use an innovative rest seat design was based on a desire to enhance retention, stability, and esthetics without relying on conventional clasping. Convergent abutments generally offer more favorable conditions for prosthesis fabrication due to their inherent mechanical advantage in providing a single path of insertion and greater retention. By contrast, divergent abutments present several clinical challenges, including poor seating of the prosthesis, decreased retention due to lifting forces, compromised marginal adaptation, and an increased risk of long-term prosthetic failure. 

When confronted with divergent abutments, modifications to the preparation are typically the first-line solution, ideally reducing axial wall angulation to achieve a total occlusal convergence of 5°-10°. Where re-preparation is not feasible due to anatomical or structural limitations, additional retention features such as axial grooves, boxes, or dovetail modifications (as demonstrated in this case) can be employed. Modified cementation protocols involving adhesive resin cements and mechanical retention aids may further enhance stability. In severe cases, alternative prosthetic designs such as sectional prostheses, telescopic crowns, or implant-supported restorations may be considered. Thus, while the current case involved relatively favorable abutment geometry, the technique described remains clinically relevant, especially in scenarios where divergence cannot be corrected or clasping is esthetically unacceptable.

A technique previously described by Jamani and Fayyad involved an indirect-direct approach using a two-part provisional FPD stabilized by a commercially available stainless steel wrought-wire clasp in combination with an occlusal rest [3]. While effective in achieving positional stability and distributing occlusal loads, the visible clasp component raises esthetic concerns, particularly in cases involving anterior or premolar teeth [9,10]. Furthermore, although the authors prepared a triangular rest seat, they acknowledged that this design did not prevent horizontal stabilization, highlighting a mechanical limitation in the technique.

By contrast, the technique presented in the present case builds upon the indirect-direct philosophy but offers distinct and clinically significant improvements. Instead of relying on a visible clasp, a rest seat with a dovetail configuration is prepared on the provisional crown fabricated on the cast. This unique design provides dual stabilization, resisting both lateral and proximal displacement, without compromising esthetics [11]. The dovetail geometry introduces near-vertical walls and internal undercuts that allow mechanical interlocking of the second segment of the provisional prosthesis while maintaining a seamless external appearance [12].

In addition, rather than sectioning the prosthesis or inserting metal components externally, the technique incorporates a wrought wire internally within the rest seat. This wire is customized and stabilized during fabrication, and then reinforced intraorally with bis-GMA composite. This not only improves mechanical retention but also ensures a smooth, hygienic, and aesthetically pleasing final outcome [13,14]. The absence of visible metal and the monolithic reinforcement design allow the restoration to blend naturally with adjacent dentition and enable easier plaque removal, particularly important in long-term provisionalization or in medically compromised patients.

Another unique advantage of this technique is the sequencing of fabrication. The initial crown is fabricated only on one abutment, which allows precise control of rest seat placement and alignment before proceeding with the second segment. This eliminates the need to section the prosthesis to manage path divergence and instead uses planned reinforcement to achieve a unified and retentive two-part restoration.

The presented technique requires extra preparation of a rest seat on the abutment tooth (35 in the present case). This rest seat is designed to provide both vertical support and resistance to horizontal displacement of the prosthesis. To achieve adequate resistance, the depth of the rest seat often needs up to 1.5 mm. To achieve resistance to horizontal displacement, the depth of the rest seat may need to be increased. This might cause pulp exposure during preparation, which is a limitation of the technique. Alternatively, to overcome this drawback, the preparation can be extended slightly over the central fossae for dovetail preparation while keeping the depth the same.

Although the technique demands precise execution, particularly in designing the rest seat to avoid over-preparation and in accurately adapting the wrought wire to fit passively, it ultimately minimizes the need for extensive intraoral modifications. By eliminating visible clasps and incorporating internal reinforcement, it provides an aesthetically pleasing and easy-to-clean provisional restoration, especially suitable for patients with high aesthetic demands or hygiene concerns. It is best suited for situations involving tilted or nonparallel abutments where esthetic demands are high, or where clasping may not be acceptable to the patient [10,15]. It may also benefit geriatric or medically compromised patients by reducing the need for prolonged chairside adjustments [16].

One potential drawback of this technique is the difficulty in retrieving the provisional FPD without dislodging or damaging the reinforced rest seat or composite seal, especially if excess cement or undercuts are present. Care must be taken during fabrication to ensure passive fit, precise adaptation of the wrought wire, and avoidance of locking features that could complicate removal. Additionally, the provisional should be luted with a weak, non-eugenol cement to facilitate easy removal without compromising the underlying tooth structure. Another limitation of the presented technique is the need for additional tooth preparation to create the rest seat. Achieving adequate depth for horizontal and proximal resistance may risk pulp exposure, especially in teeth with large pulp chambers or reduced coronal height, and thus must be approached with caution and pre-evaluation using radiographs.

While promising, this technique must be applied selectively and tailored to the clinical situation. Controlled studies comparing its biomechanical performance, patient satisfaction, and durability against traditional clasp-retained methods would further support its utility in routine clinical practice. Overall, the design allows for multidirectional stabilization while maintaining a monolithic, aesthetic external surface. It also ensures functional retention, facilitates oral hygiene, and enables minimally invasive intraoral adjustments during the final tooth preparation phase.

## Conclusions

The described technique offers a conservative, esthetically driven approach for provisional FPD fabrication in cases with nonparallel abutments. By eliminating visible clasping and incorporating internal reinforcement within a dovetail-configured rest seat, it may enhance retention and patient comfort while maintaining hygiene and esthetics. Although promising, this technique should be considered a viable alternative in carefully selected cases, and further clinical studies are warranted to evaluate its long-term effectiveness and broader applicability.
